# A retrospective study evaluating the correlation between the severity of intervertebral disc injury and the anteroposterior type of thoracolumbar vertebral fractures

**DOI:** 10.6061/clinics/2016(06)02

**Published:** 2016-06

**Authors:** Yunshan Su, Dong Ren, Yan Zou, Jian Lu, Pengcheng Wang*

**Affiliations:** Third Hospital of Hebei Medical University, Orthopaedic Trauma Service Center, Major Laboratory of Orthopaedic Biomechanics in Hebei Province, Shijiazhuang, China

**Keywords:** Intervertebral Disc, Injury, Thoracolumbar Vertebra, Fracture, Correlation

## Abstract

**OBJECTIVE:**

To evaluate the correlation between the severity of intervertebral disc injury and the anteroposterior type of thoracolumbar vertebral fractures.

**METHODS:**

Fifty-six cases of thoracolumbar vertebral fractures treated in our trauma center from October 2012 to October 2013 were included in this study. The fractures were classified by the anteroposterior classification, whereas the severity of intervertebral disc injury was evaluated using magnetic resonance imaging. The Spearman correlation coefficient was used to analyze the correlation between the severity of intervertebral disc injury and the anteroposterior type of thoracolumbar fractures, whereas a *χ*^2^ test was adopted to measure the variability between different fracture types and upper and lower adjacent disc injuries.

**RESULTS:**

The Spearman correlation coefficients between fracture types and the severity of the upper and lower adjacent disc injuries were 0.739 (*P*_U_<0.001) and 0.368 (*P*_L_=0.005), respectively. It means that the more complex Arbeitsgemeinschaft für Osteosynthesefragen (AO) classifications are the disc injury is more severe. There was also a significant difference in the severity of injury between the upper and lower adjacent discs near the fractured vertebrae (*p*<0.001).

**CONCLUSIONS:**

In thoracolumbar spinal fractures, the severity of the adjacent intervertebral disc injury is positively correlated with the anteroposterior fracture type. The injury primarily involves intervertebral discs near the fractured end plate, with more frequent and severe injuries observed in the upper than in the lower discs. The presence of intervertebral disc injury, along with its severity, may provide useful information during the clinical decision-making process.

## INTRODUCTION

Thoracolumbar vertebral fractures are usually complicated by varying degrees of intervertebral disc injury 1,2. Clinicians often make a clinical decision based on the degree of spinal stability and the extent of neurological damage. However, spinal stability is primarily assessed based on the fracture type and the integrity of the posterior ligamentous complex, whereas the presence of intervertebral disc injury is frequently ignored. Intervertebral disc injury is an important factor that influences postoperative spinal stability and prognosis 3,4. However, the common clinical classification systems for thoracolumbar vertebral fractures, including the AO classification, Denis classification, load-sharing classification and thoracolumbar injury classification and severity score (TLICS), have not provided an adequately detailed description for the severity of intervertebral disc injury. Magnetic resonance imaging (MRI) is a reliable technique for evaluating intervertebral disc injury. Sander et al. 5 proposed a novel classification system for the severity of intervertebral disc injury based on the MRI images of 204 discs from 102 patients. However, a correlation between the severity of intervertebral disc injury and the AO fracture type was not identified. Therefore, the main goal of our study was to determine whether such a correlation exists.

## MATERIALS AND METHODS

The X-ray, computerized tomography (CT) and MRI data of patients with traumatic thoracolumbar vertebral fractures (T11–L2) admitted to the trauma center between October 2012 and October 2013 were analyzed retrospectively. The inclusion criteria were patients with single segmental thoracolumbar vertebral fractures who were between 18 and 65 years old. The exclusion criteria were patients with incomplete imaging data or with evidence of spinal deformities, spinal tumors, spinal tuberculosis, discitis, intervertebral disc degeneration, bone metabolism disease and osteoporotic compression fractures. Ultimately, 56 patients were included in this study.

Using the anteroposterior and lateral X-ray and CT images, two senior surgeons (Sys and Rd) confirmed the fracture types based on the AO classification system 3 and verified the surgical regimens in combination with the MRI data. Adopting the classification system for intervertebral disc injury proposed by Sander et al., 5 upper and lower adjacent disc injuries were divided into four grades from Grade 0 to Grade 3 based on the MRI data. Patients with Grade 2 and Grade 3 injuries were classified as having an intervertebral disc injury. All radiographs were independently read by two senior surgeons (WPc and Lj) who were blinded to the patient data. Sagittal T1 and T2 weighted images (T1WI, T2WI) and Turbo inversion recovery magnitude (TIRM) MR images were acquired by the 1.5T clinical MRI system (Magnetom Avanto TIM, Siemens Medical Solutions, Erlangen, Germany), and the specific sagittal scanning parameters were as follows: field of view 32 cm; matrix 512 * 256; slice thickness 4 mm; T1WI: TR624, TE11; T2WI: TR3500, TE87; and TIRM: TR3500, TE30, and TI160.

If their conditions permitted, combined anterior and posterior surgeries were performed for the patients suffering from incomplete neurological damage caused by anterior vertebral elements (e.g., compressions) and severe intervertebral disc injuries, whereas the other patients were treated by posterior short-segment pedicle instrumentation.

Clinical statistical data were analyzed by the IBM SPSS Statistics 22.0. The kappa value was used to assess intra- and inter-observer agreement, with *κ*≥0.7 indicating excellent agreement, 0.75>*κ*≥0.4 indicating passable agreement, and *κ*<0.4 indicating poor agreement. The Spearman correlation coefficient between the severity of intervertebral disc injury and the AO fracture type was calculated. A chi-square (*χ*^2^) test was adopted to measure the variability between different fracture types and between upper and lower adjacent disc injuries. The alpha level was set at 0.05 (*α*=0.05), and *p*<0.05 indicated a significant difference.

## RESULTS

Of the 56 patients, 43 were male and 13 were female. The mean age was 40.94±12.47 years (range 15–61 years). The mechanisms of injury included the following: 21 cases of falls from heights, 22 cases of traffic accidents, 10 cases of fall-related injuries and 3 cases of crush injuries caused by a heavy weight. The fractures were found at T11 (5 cases), T12 (11 cases), L1 (28 cases) and L2 (12 cases). According to the American Spinal Injury Association (ASIA) classification, neurological damage was classified as Grade A (3 cases), Grade B (1 case), Grade C (0 case), Grade D (15 cases) and Grade E (37 cases); there were 12 cases of AO type-A1 fracture, 2 cases of AO type-A2 fracture, 38 cases of AO type-A3 fracture, 1 case of AO type-B1 fracture, 2 cases of AO type-B2 fracture and 1 case of AO type-C3 fracture.

Regarding the severity of injuries in the upper adjacent discs, 1.8% (1/56) of the cases were Grade 0, 16.1% (9/56) were Grade 1, 12.5% (7/56) were Grade 2, and 69.6% (39/56) were Grade 3 (*κ*=0.853). Regarding the severity of injuries in the lower adjacent discs, 25% (14/56) of the cases were Grade 0, 44.6% (25/56) were Grade 1, 23.2% (13/56) were Grade 2 and 7.1% (4/56) were Grade 3 (*κ*=0.894). Excellent intra- and inter-observer agreement was observed. With an overall rate of 56.3% (63/112), the injury rate of intervertebral disc injury (number of patients with Grade 2 and Grade 3 injuries) was 82.1% (46/56) in the upper adjacent discs and 30.2% (17/56) in the lower adjacent discs.

Table 1 lists the severity of the disc injuries and the distribution of the fracture types. The Spearman correlation coefficient between the severity of upper and lower adjacent disc injuries and the AO fracture types was 0.739 and 0.368, respectively, which was significant (*P*_U_<0.001 and *P*_L_=0.005, respectively) and indicated a positive correlation between the two parameters.

The results of the *χ*^2^ test for differences between the AO fracture types and the severity of both upper and lower adjacent disc injuries were significant (*P*_U_<0.001, *P*_L_=0.003), indicating a difference in the severity of disc injuries among different AO fracture types. The *χ*^2^ test also showed a significant difference in severity between upper and lower adjacent disc injuries (*p*<0.001, Table 2), indicating that the upper disc was more susceptible to injuries and was associated with more severe injuries than the lower disc.

## DISCUSSION

This study indicates that the degree of intervertebral disc injury increases with the severity of the fracture and that intervertebral disc injury is positively correlated with the AO fracture type. To the best of our knowledge, this is the first report demonstrating such correlations. These findings remind us that, in addition the characteristics of the vertebral fractures, clinicians should pay attention to the accompanying intervertebral disc injuries during the clinical decision-making process. Moreover, the results also demonstrate the need to integrate the assessment of intervertebral disc injury into the classification system for thoracolumbar vertebral fractures.

Thoracolumbar vertebral fractures in young adults are common and are often associated with profound social consequences. Most of these primarily affect males and result from motor accidents and falls from heights, which are associated with high kinetic energy 6. The intervertebral disc is a cartilage complex that is vulnerable to injury from stretches or shear force, whereas great compression forces can also result in traumatic intervertebral disc injuries 7. Of the 56 patients included in this study, 82.1% (46/56) suffered from intervertebral disc injuries, revealing the vulnerability of intervertebral discs during thoracolumbar vertebral fractures. Thoracolumbar vertebral fractures are often caused by axial compression forces and may also be associated with flexion distractions, torsions and shear forces, making intervertebral discs susceptible to injuries during fracture.

**Figure 1:**
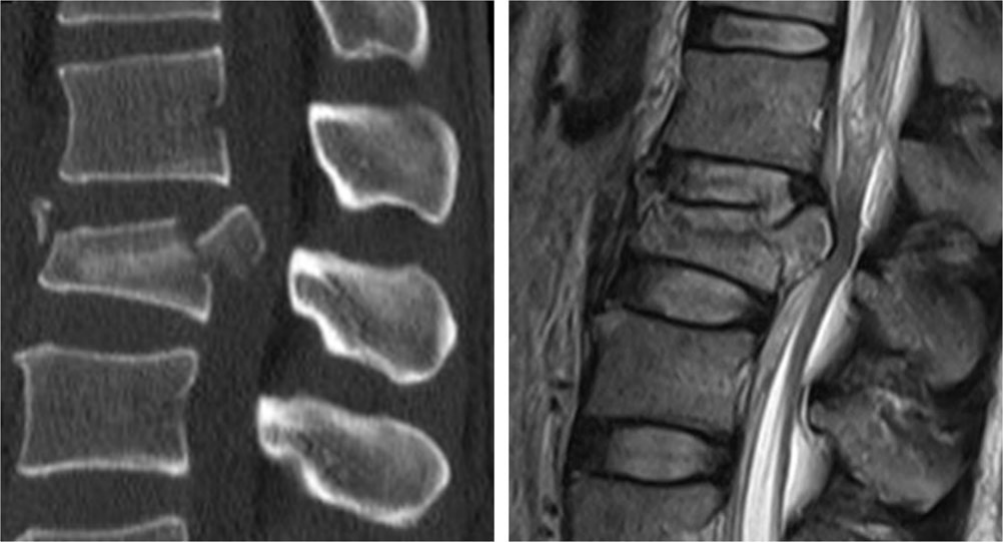
36-year-old man with an A2.2 fracture at level L1. T2WI magnetic resonance image shows that both the upper and the lower intervertebral discs are injured at a Grade 3 level.

**Figure 2:**
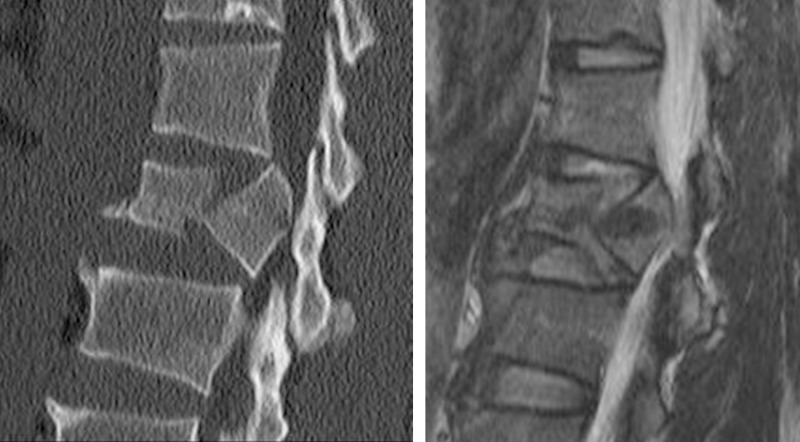
34-year-old man with an A3.1 fracture at level L2. T2WI magnetic resonance image shows that the upper intervertebral disc is injured at a Grade 3 level and that the lower disc is a Grade 1 injury.

**Figure 3:**
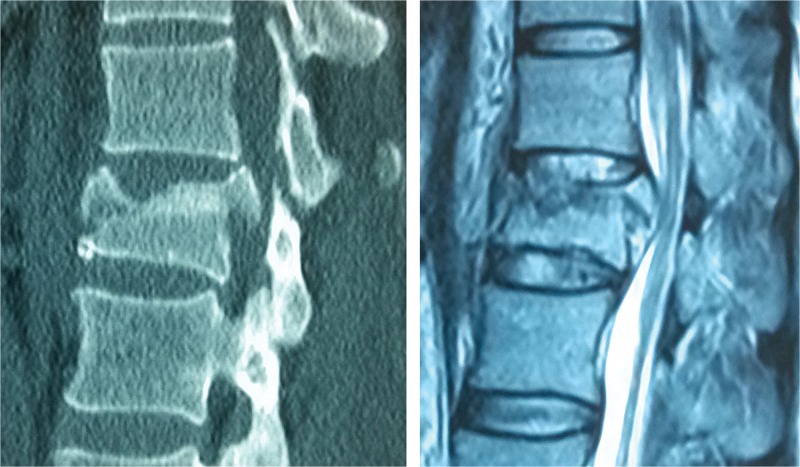
44-year-old woman with an A3.2 fracture at level L1. T2WI magnetic resonance image shows that the upper intervertebral disc is injured at a Grade 3 level and that the lower disc is a Grade 2 injury.

Oner et al. employed MRI to examine cadaver specimens with thoracolumbar vertebral fractures and disc injuries and confirmed that MRI was reliable in assessing acute intervertebral disc injury 8. In addition, they also reclassified the disc changes into 6 types based on the MRI results of 75 thoracolumbar vertebral fractures, and they noted that the occurrence of thoracolumbar vertebral fractures complicated by intervertebral disc injuries was not uncommon. Furthermore, they suggested that the traditional AO classification system was not appropriate for describing patients with spinal fractures accompanied by intervertebral disc injuries 1,8. Sander et al. 5 proposed a more detailed classification system with excellent intra- and inter-observer agreement (*κ*=0.96). We therefore adopted this classification system as the assessment criteria in this study and evaluated the severity of intervertebral disc injuries based on the preoperative MRI images. Roaf 9 noted that the intervertebral disc is stronger than the vertebral end plate under compression forces and that compression forces will result in fractures of the vertebral end plate before the intervertebral disc is injured. Valentini et al. 10 proposed that the protrusion of the injured intervertebral disc nucleus pulposus into the vertebral body is the mechanism underlying an unstable burst fracture. Based on the results of the present study, injury to the intervertebral disc often occurs to the discs adjacent to the fractured end plate. Moreover because the upper vertebral end-plate fracture is more frequent than the lower vertebral end-plate fracture, the upper intervertebral disc is more susceptible to injury than the lower intervertebral disc. According to the results of our study, the proportion of intervertebral disc injuries was small and the severity was mild in patients with AO type-A1 fractures. Both upper and lower adjacent disc injuries were observed in patients with AO type-A2 fractures (Figure 1: 36-year-man with an A2.2 fracture at level L1. T_2_WI MR image shows that both the upper and the lower intervertebral discs are injured at a Grade 3 level). Upper adjacent disc injuries were found in all the patients with AO type-A3 fractures (Figure 2:34-year-man with an A3.1 fracture at level L2. T_2_WI MR image shows that the upper intervertebral disc is injured at a Grade 3 level and that the lower disc is a Grade 1 injury), whereas lower adjacent disc injuries were also found in some of the patients with AO type-A3.2 (Figure 3: 44-year-woman with an A3.2 fracture at level L1. T_2_WI MR image shows that the upper intervertebral disc is injured at a Grade 3 level and that the lower disc is a Grade 2 injury) and type-A3.3 fractures. Vertebral burst fractures and upper adjacent disc injuries were observed in all patients with AO type-B and type-C fractures. Lower adjacent disc injuries were found in all patients with AO type-B1 and C3 fractures. Additionally, injuries in patients with AO type-A2, A3, B1, B2, and C3 fractures were more severe than those in patients with AO type-A1 fractures. Moreover, it was also noted that signal changes (Grade 1) may occur in the adjacent discs before the vertebral end-plate fracture, with injuries to the upper disc being more severe.

The intervertebral disc is a part of the passive stabilizing subsystem of the spine. Both damage and degeneration of the intervertebral disc can affect the function of the passive stabilizing system of the spine 11. In a biomechanical study, Lin et al. 3 found that the vertebra, upper adjacent disc and lower adjacent disc accounted for 38%, 35% and 27% of all of the unstable factors after thoracolumbar burst fractures, respectively. However, controversy remains regarding the level of severity of an intervertebral disc injury that requires surgical intervention. Wang et al. 12 compared MRI data before and after percutaneous pedicle screw fixation and found that, in thoracolumbar burst fractures, disc degeneration was strongly associated with end plate fracture, particularly fracture of the upper adjacent end plate. Shi et al. 13 conducted a minimum 7-year follow-up of 52 patients who had undergone posterior pedicle screw instrumentation for thoracolumbar fractures; they reported that postoperative correction loss was primarily caused by the loss of intervertebral disc space height of the upper and lower discs adjacent to the injured vertebra. Furthermore, there were greater losses observed in the upper discs than in the lower discs and the height losses of the fractured vertebra were relatively smaller. During burst fractures, the injured intervertebral disc tissues have been reported to protrude into the vertebral body through the fractured end plate, and posterior pedicle screw instrumentation can indirectly restore the peripheral end plate via the attached annulus fibrosus. However, the center of the end plate is in a compression state and develops a cup-like deformity because it is not attached by the annulus fibrosus. Under such conditions, the protruded intervertebral disc tissues result in a great loss of intervertebral space height [Bibr b14-cln_71p297][Bibr b15-cln_71p297]-16. In our study, if their conditions permitted, combined anterior and posterior surgeries were performed for patients suffering from incomplete neurological damage caused by anterior vertebral elements (e.g., compressions) and severe intervertebral disc injuries. However, for patients with severe fractures and intervertebral injuries, an agreement has not yet been reached on whether an anterior surgery, discectomy and vertebral resection for the injured discs and vertebrae, and vertebral interbody fusion are required.

As this study was a retrospective analysis of patient imaging data, innate limitations are inevitable. This was a single-center study with a small sample size, and confirmation of our findings from prospective studies involving multiple centers and a large sample size is required. We are performing further research to determine the operative indications for injured discs by comparing the MRI data before and after pedicle screw fixation (without injured disc intervention) and assessing the long-term results of this intervention. Clinicians should take note of any associated intervertebral disc injuries to decide on the optimal surgical strategy.

In conclusion, the severity of the adjacent intervertebral disc injury is positively correlated with the AO fracture type during thoracolumbar spinal fractures. The injury primarily involves intervertebral discs near the fractured end plate, with more frequent and severe injuries observed in the upper discs than in the lower discs. The existence of intervertebral disc injury and its severity may provide useful information during the clinical decision-making process.

## AUTHOR CONTRIBUTIONS

Wang P conceived and designed the study. Su Y wrote the manuscript and participated in data management. Ren D collected and processed the clinical data. Zou Y and Lu J performed the statistical analysis.
